# Designing Splicing Digital Microfluidics Chips Based on Polytetrafluoroethylene Membrane

**DOI:** 10.3390/mi11121067

**Published:** 2020-11-30

**Authors:** Haoqiang Feng, Zichuan Yi, Ruizhi Yang, Xiaofeng Qin, Shitao Shen, Wenjun Zeng, Lingling Shui, Guofu Zhou, Chongfu Zhang

**Affiliations:** 1 College of Electron and Information, University of Electronic Science and Technology of China, Zhongshan Institute, Zhongshan 528402, China; haoqiang.feng@m.scnu.edu.cn (H.F.); zwjcareer@163.com (W.Z.); Shuill@m.scnu.edu.cn (L.S.); cfzhang@uestc.edu.cn (C.Z.); 2 South China Academy of Advanced Optoelectronics, South China Normal University, Guangzhou 510006, China; yangruizhi2015@163.com (R.Y.); xiaofengqin@m.scnu.edu.cn (X.Q.); shenshitao@m.scnu.edu.cn (S.S.); guofu.zhou@m.scnu.edu.cn (G.Z.)

**Keywords:** digital microfluidics (DMF), electrowetting-on-dielectric (EWOD), splicing gap, slippery liquid infused porous surface (SLIPS), polytetrafluoroethylene (PTFE)

## Abstract

As a laboratory-on-a-chip application tool, digital microfluidics (DMF) technology is widely used in DNA-based applications, clinical diagnosis, chemical synthesis, and other fields. Additional components (such as heaters, centrifuges, mixers, etc.) are required in practical applications on DMF devices. In this paper, a DMF chip interconnection method based on electrowetting-on-dielectric (EWOD) was proposed. An open modified slippery liquid-infused porous surface (SLIPS) membrane was used as the dielectric-hydrophobic layer material, which consisted of polytetrafluoroethylene (PTFE) membrane and silicone oil. Indium tin oxide (ITO) glass was used to manufacture the DMF chip. In order to test the relationship between the splicing gap and droplet moving, the effect of the different electrodes on/off time on the minimum driving voltage when the droplet crossed a splicing gap was investigated. Then, the effects of splicing gaps of different widths, splicing heights, and electrode misalignments were investigated, respectively. The experimental results showed that a driving voltage of 119 V was required for a droplet to cross a splicing gap width of 300 μm when the droplet volume was 10 μL and the electrode on/off time was 600 ms. At the same time, the droplet could climb a height difference of 150 μm with 145 V, and 141 V was required when the electrode misalignment was 1000 μm. Finally, the minimum voltage was not obviously changed, when the same volume droplet with different aqueous solutions crossed the splicing gap, and the droplet could cross different chip types. These splicing solutions show high potential for simultaneous detection of multiple components in human body fluids.

## 1. Introduction

Since the concept of the micro total analysis system (μ-TAS) was put forward in the 1990s [1], microfluidic technology has developed rapidly. With the gradual maturity of the technology, more and more experiments or tests have been executed on lab-on-a-chip (LOC) [2,3]. This technology has the advantages of reducing sample volume, fast reaction rate, and integration capabilities, etc. [4]. Therefore, this technology has been widely used in many fields, such as cell-based analysis, enzyme linked immunosorbent assay (ELISA), and point-of-care (PTOC), etc. [5,6,7]. Microfluidics can be divided into the following two categories: continuous microfluidics and discreet droplet microfluidics. Continuous microfluidics is suitable for some basic or long-defined simple applications, however, discreet droplet microfluidics can allow for independent control of each droplet [8]. Digital microfluidics (DMF) is a kind of discrete droplet microfluidics that realizes on the miniaturization of a device, and also realizes on the precise control of individual droplets with a high degree of automation [9,10,11]. Because of these advantages, the technology has been widely used in fields of biochemical experiments, medical testing, and optical applications, etc. [12,13,14,15].

It is necessary to integrate some functional components on a DMF equipment, such as micro-heating components, pumping components, and sensors, etc. [16,17,18]. This can provide better conditions for complex experiments [19,20,21]. Although some people have done some research on function integration, they have not yet developed a truly multifunctional chip. For instance, Srinivasan V et al. [22] proposed a chip construction scheme for integrating multiple applications on a single chip device, and successfully detected the glucose measurement of human physiological fluid. This solution could achieve a high level of integration and automation. However, the dilution range of the sample was too small, which made some experiments impossible to complete on this chip. Shamsi M.H et al. [23] proposed a method to integrate an electrochemiluminescence method on a DMF chip to detect microRNA. It could solve the cumbersome steps in RNA analysis, and be applied to other areas, such as food, water quality testing, immunoassays, etc. [24]. However, the proposal of this solution discarded the original droplet separation function of the chip, and the design of the electrode was greatly restricted. Jain et al. [25] and Yi et al. [26] tried to use different substrate materials to make DMF chips. However, due to the limitation of its manufacturing process, the electrode could not reach the height of indium tin oxide (ITO) glass chip, and therefore the choice of the dielectric layer had great restrictions. In recent reports, paper-based DMF chips such as photographic paper have been manufactured with greatly reduced manufacturing costs. In addition, it had the characteristic of flexibility, which was a good prospect for the development of DMF technology [27,28], but it was not as convenient as the ITO glass when a dielectric layer was used. In addition, connection methods between chips were proposed by Abdelgawad et al. [29], Moazami et al. [30], and Michael et al. [31], respectively. However, few people have studied the splicing of open DMF chips.

In this paper, a splicing open DMF system based on ITO glass was proposed. An open modified slippery liquid infused porous surface (SLIPS) membrane was used as the dielectric-hydrophobic layer material, which could overcome the obstacle of the gap between two chips. In experiments, first, we investigated the effect of the electrode on/off time of crossing the splicing gap for a droplet. Then, we investigated the relationship between the minimum driving voltage for driving droplets and different splicing solutions. Finally, different materials of the droplet could cross the splicing gap. The system showed good universality for various experiments. In addition, the droplet could cross different chips, such as PCB-based chips, which realized integration of functions.

## 2. Principle

There are many driving mechanisms for the DMF, such as electrowetting (EW) [32], electrowetting-on-dielectric (EWOD) [33], dielectrophoresis (DEP) [34], surface acoustic wave (SAW) [35], optoelectrowetting (OEW) [36], etc. Among these mechanisms, EWOD has become the mainstream mechanism of driving droplets due to the advantages of easy operation, easy processing, simple peripheral control circuits, and low cost [37]. Here, the EWOD mechanism was used as the theory and dynamics of droplet movement, which was very important for fabrication of chips. According to the well-known Young–Lippman equation [38], expressed as Equation (1), when the applied driving voltage is limited, the thinner the thickness of the dielectric layer and the larger the dielectric constant, the greater the change in the contact angle of the droplet. Therefore, the droplet can be driven at a smaller voltage.

(1) $$\cos\theta_{V}=\cos\theta_{0}+\frac{\varepsilon_{0}\varepsilon_{r}V^{2}}{2\gamma_{lg}d}$$

where *θ*_0_ and *θ_V_* represent the contact angle before and after voltage application, respectively; *ε*_0_ and *ε_r_* are the dielectric constant in vacuum and the effective dielectric constant of the dielectric layer, respectively; *V* is the driving voltage; *d* is the thickness of the dielectric layer; and *γ_lg_* is the surface of the liquid-gas interface tension.

**Figure 1:**
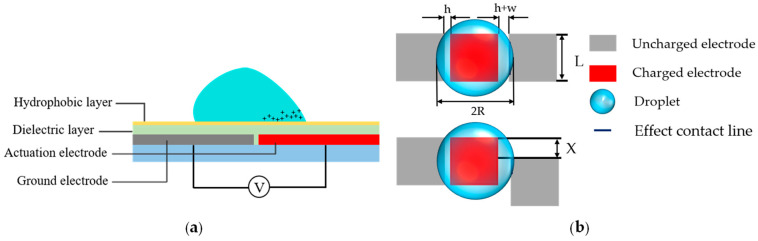
(**a**) Schematic diagram of driving a droplet on an open electrowetting-on-dielectric (EWOD) chip; (**b**) Schematic diagram of the effective contact line of a droplet when the electrode is normal and misaligned.

Droplet movement is driven by the principle of EWOD. As shown in Figure 1a, if a droplet can be driven successfully, it must touch the next electrode, otherwise, there is no electrowetting force which can act on the droplet. The movement of a droplet is caused by the potential difference between two adjacent electrodes. When a high voltage is applied to one side of the droplet, the charge can accumulate on the surface of the droplet. Then, a certain value of the charge is reached, and the shape of the droplet is changed irregularly. At this time, a pressure difference is formed inside the droplet to drive it to move [39]. Here, electrowetting force is expressed as an effective contact line length between the droplet and the driving electrode. As shown in Equation (2), the longer the effective contact line, the greater the electrowetting force on the droplet [40]. For a drop of the same volume, the minimum driving force is certain. In this paper, it is assumed that the droplet deforms circularly on the electrified electrode, the radius of the droplet is *R*, the electrode side is *L*, the spacing of electrodes is h, and the width of the splicing gap is *w*, as shown in Figure 1b. According to geometric calculation, we get the relationship between the effective contact line and the electrode misalignment, as shown in Equation (3). When the misalignment between two adjacent electrodes becomes larger and larger, the length of the effective contact line remains unchanged at first, and then becomes smaller as the misalignment becomes larger. Therefore, the driving voltage for droplet movement does not change at first, and then increases. *N* is the effective contact line when there is no misalignment, *M* is half the length of the electrode side that is not covered by the droplet when there is no electrode misalignment, *S* is the length where the electrode misalignment just has no effective contact line, and *θ* is an angle introduced according to calculation needs. These values are shown in Equations (4)–(7).

(2) $$F_{x}=L_{eff}\gamma \left(\cos\theta_{V}-\cos\theta_{0}\right)=\frac{\varepsilon_{0}\varepsilon_{r}L_{eff}V^{2}}{2d}$$

(3) $$L_{eff}=\{\begin{array}{c} 2N;\;X\in \left[0,M\right]\\ \sqrt{{\left[2N-\left(X-M\right)\right]}^{2}+{\left[N-\left(X-M\right)-\left(\frac{L}{2}+h+w\right)\tan\theta \right]}^{2};X\in \left(M,S\right)} \end{array}$$

(4) $$N=2\sqrt{R^{2}-{\left(\frac{L}{2}+h+w\right)}^{2}}$$

(5) $$M=\frac{L}{2}-\sqrt{R^{2}-{\left(\frac{L}{2}+h+w\right)}^{2}}$$

(6) $$S=\frac{L}{2}+\sqrt{R^{2}-{\left(\frac{L}{2}+h+w\right)}^{2}}$$

(7) $$\theta =\sin^{-1}\frac{N-\left(X-M\right)}{R}$$

where $F_{x}$ is electrowetting force, $L_{eff}$ is the effect contact line, γ is interfacial tension, and *X* is a value of electrode misalignment.

## 3. Fabrication of the Digital Microfluidics (DMF) System

**Figure 2:**
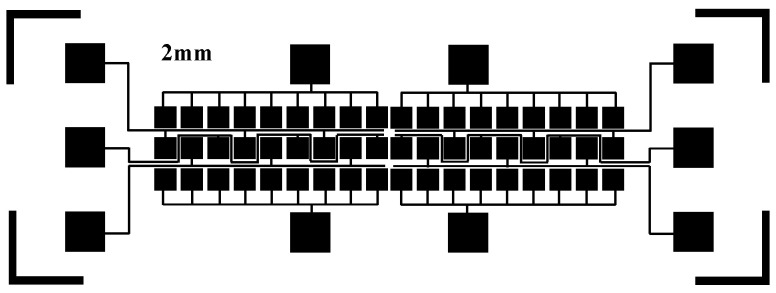
The electrode array of a digital microfluidics (DMF) chip, the size of a square electrode is 2 × 2 mm^2^, and the gap between two electrodes is 0.2 mm.

The manufacture of a DMF chip includes a substrate, an electrode array, a dielectric layer, and a hydrophobic layer. In order to investigate the performance of the splicing DMF chips for droplets, we used an ITO glass as the substrate material for a DMF chip, and the photolithography process was used for making electrode arrays. The process mainly included the following steps: glass clearing, heating, spinning, former baking, exposing, developing, latter baking, and etching [41]. The detailed process steps were submitted in the support materials. Considering problems of chip manufacturing, the electrode shape was designed as a square electrode with a size of 2 × 2 mm^2^ and the gap between two electrodes was designed to be 0.2 mm. The electrode array is shown in Figure 2, where two sides were ground electrodes, and the middle was a driving electrode. This electrode array was designed by AutoCAD software (2019, Autodesk, San Rafael, CA, USA).

**Figure 3:**
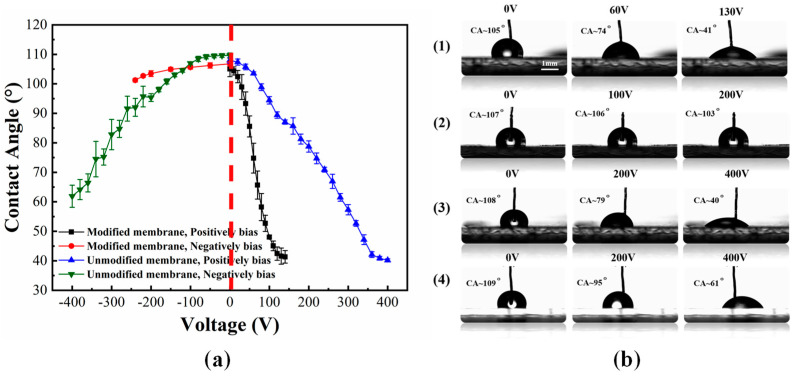
Electrowetting performance of a polytetrafluoroethylene (PTFE) membrane before and after modification by a 10 μL droplet. (**a**) Curve of contact angle varying with the applied voltage; (**b**) Microscope images of contact angle measurement of droplets on a modified PTFE membrane and an unmodified PTFE membrane; (**1**) Microscope measurement of the contact angle of a modified PTFE membrane with different positive biases; (**2**) Microscope measurement of the contact angle of a modified PTFE membrane with different negative biases; (**3**) Microscope measurement of the contact angle of an unmodified PTFE membrane with different positive biases; (**4**) Microscope measurement of the contact angle of an unmodified PTFE membrane with different negative biases.

**Figure 4:**
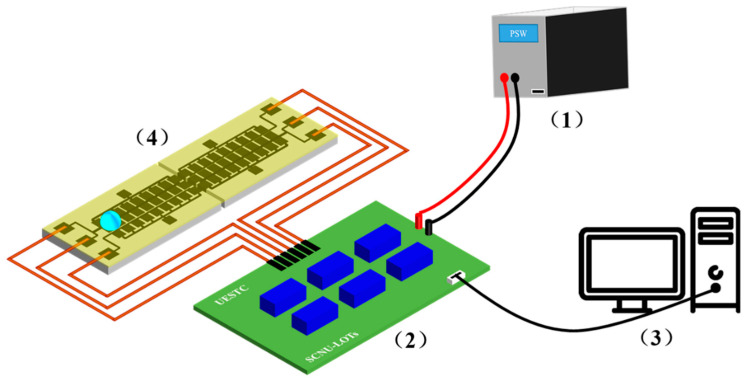
Schematic of the DMF system. (**1**) DC source; (**2**) a relay; (**3**) a computer; (**4**) a splicing DMF chip.

The dielectric-hydrophobic layer has a significant influence on the performance of a DMF chip. In order to simplify the complicated process flow for preparing a dielectric-hydrophobic layer, an open modified SLIPS membrane was used as the dielectric-hydrophobic layer [42,43]. We utilized a continuously thin polytetrafluoroethylene (PTFE) membrane which was modified with 1H,1H,2H,2H-perfluorooctyltrichlorosilane (PFOTS). The PTFE membrane (composed of nanofibrous networks, ~23 μm thick, average pore size ~200 nm, dielectric constant ~2.1) had good stability [44,45]. According to our previous experiments, there was no significant difference in the moving speed of a droplet when a PTFE membrane was placed for 28 days, and it could be cycled thousands of times and stored for a long time [46]. The manufacturing steps of the SLIPS modified PTFE membrane were as follows: Firstly, we cut a PTFE membrane according to the size of the electrode array. Then, the membrane was soaked in 0.03 %wt PFOTS ethanol solution for one hour, and then, it was taken out and pasted on a spliced DMF chip. Secondly, the DMF chip was placed on an ultraclean table (SW-CJ-2FD, ShanghaiBOXUN, Shanghai, China) and the ethanol was evaporated completely. After evaporation of the ethanol, 5 cst silicone oil was infused into the modified PTFE membrane to form a SLIPS. In our study, 10 μL of silicone oil was injected into 1 × 1 mm^2^ of modified PTFE membrane, and the membrane was placed horizontally for 10 min, the silicone oil was spread by capillarity. The membrane was placed vertically for 2 h, and then it was placed horizontally for 30 min. The whole process was operated on an ultraclean table. We tested the electrowetting performance of the modified PTFE membrane using a contact angle measuring instrument (OCA15pro, Dataphysics Instruments Gm6H, Filderstadt, Germany), as shown in Figure 3a,b. As compared with an unmodified membrane, the required voltage was significantly reduced, when the droplet’s contact angle was changed to the same degree. However, there was a significant difference in the degree of change in the contact angle between positive bias and negative bias. With positive bias, only low voltages were required to change the contact angle of a droplet, but with negative bias, voltages were increased to 250 V, the droplet contact angle degree was changed less than 10 degrees. Therefore, we could apply different polarity voltages to adjacent electrodes for driving a droplet. In order to investigate the effect of a splicing gap size on the movement of droplets, we fabricated splicing gap chips of different sizes. Here, we utilized a wafer dicing machine (DS610, Shenyang Heyan Technology Co., Ltd., Shenyang China) to cut an ITO glass chip with an established path. Then, a long tail clip, a double-sided tape, and a glass substrate were used to make DMF chips. Spacers whose thickness was 50, 100, and 500 μm, respectively, were used to characterize splicing width, splicing height difference, and electrode misalignment difference, respectively (as shown in Figure S1). The average error sizes were 0.82%, 5.55%, and 0.80%, respectively, by a detailed calculation. These errors were in an acceptable range, and this kind of characterization scheme was feasible. Finally, the connection schematic diagram of the whole system is shown in Figure 4. The program of the droplet movement path was edited by the C programming language with a software (uVision5, Keil, Cambridge, UK, USA). Then, the program was burned into the relay through the USB interface. DC source (PSW-800-1.44 Gwinstek, Taiwan, China) was used to provide driving force, and the voltage signal was outputted one by one with a relay.

## 4. Results and Discussions

### 4.1. Effect of the Electrode On/Off Time

**Figure 5:**
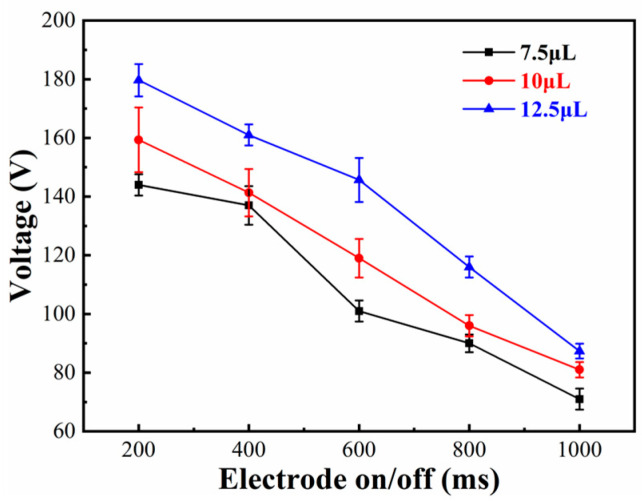
Curves of the relationship between the minimum driving voltage and the different electrode on/off times in an open EWOD device.

To investigate the effect of the electrode on/off time on the droplet crossing the splicing gap, we set 200, 400, 600, 800, and 1000 ms as on/off times, respectively. The splicing gap between two chips was fixed at 300 μm, and the minimum driving voltages of a droplet for crossing the splicing gap with different electrode on/off times were measured. As shown in Figure 5, a different volume of droplets was driven in the same condition. The minimum driving voltage of the droplet decreased as the electrode on/off time increased. A 7.5 μL droplet could cross the splicing gap with an electrode on/off time of 1000 ms and a driving voltage of 71 V. However, with an electrode on/off time of 200 ms, 144 V was required. Therefore, the longer the on/off time was, the longer the electrowetting force that could be applied to the droplet. Due to the determination of friction and surface pinning force, the longer the action time, the smaller the required electrowetting force. In addition, the data indicated that the larger the volume of a droplet, the greater the voltage, because the resistance of a droplet was increased when the volume of a droplet was increased.

### 4.2. Effect of Different Splicing Solutions

#### 4.2.1. Effect of the Splicing Gap Width

**Figure 6:**
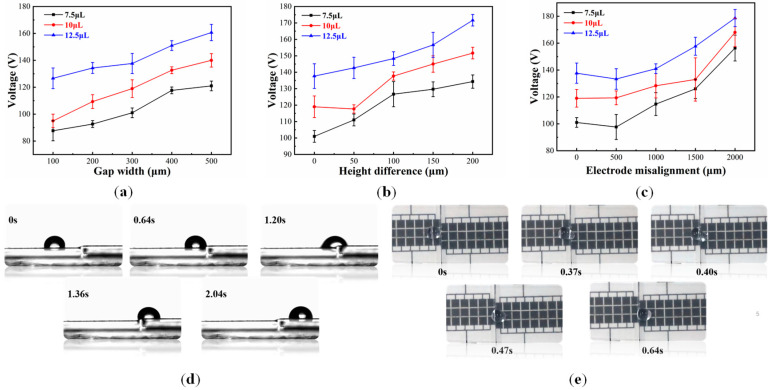
(**a**) Curves of the relationship between minimum driving voltage of droplet and gap width; (**b**) Curves of the relationship between minimum voltage required of droplet and height difference; (**c**) Curves of the relationship between minimum voltage required of droplet and misalignment difference; (**d**) A 10 μL droplet crossed a splicing gap with a height difference of 150 μm; (**e**) A 10 μL droplet crossed a splicing gap with an electrode misalignment of 1000 μm. The electrode on/off time was fixed at 600 ms, and the splicing gap width was fixed at 300 μm.

To investigate the effect of different splicing gap widths, the minimum driving voltage with different widths of the splicing gap was measured. As shown in Figure 6a, the electrode on/off time was fixed at 600 ms, and droplets’ volumes were 7.5, 10, and 12.5 μL, respectively. The curve of the splicing gap showed that the larger the splicing gap, the greater the minimum driving voltage. When the splicing gap became larger, the droplet, which was driven from the last electrode of the previous chip to the first electrode of the next chip, required a greater electrowetting force to be captured by the next electrode for crossing the splicing gap. In general, the larger the droplet volume, the greater the driving voltage for crossing a splicing gap. Interestingly, when the droplet crossed the splicing gap, the movement speed of the droplet was faster than the speed of moving on two adjacent electrodes. For example, we used the AdobepremiereproCC2019 (PrCC2019) software (2019, Adobe, San Jose, CA, USA) to analyze the moving speed of the droplets. A 10 μL droplet moved at an average speed of 6.77 mm/s between two adjacent electrodes with 105 V, while the speed was 12.86 mm/s when it moved between splicing gaps with the same voltage, which lead to the electrode array spacing producing an electrowetting force, but this electrowetting force hindered the movement of droplets. However, there was no lead distribution on both sides of the splicing gap, therefore, it could move at a higher speed.

#### 4.2.2. Effect of Height Difference of Splicing Gaps

There is a more or less height differences when two or more chips are spliced together. In order to investigate height difference effect of splicing gaps, the splicing gap was fixed at 300 μm, and the electrode time was fixed at 600 ms. Chips with height differences of 0, 50, 100, 150, and 200 μm were manufactured with 50 μm spacer, respectively. As shown in Figure 6b, the minimum driving voltage for different droplet volumes which was used to cross the splicing gap with different height differences was tested. We found that the greater the height difference, the greater the minimum driving voltage. A 10 μL droplet could cross the splicing gap with 118 V driving voltage when there was a 50 μm height difference, and it could cross the splicing gap with 145 V if the height difference was 150 μm. However, when the height difference exceeded 150 μm, the droplet could cross from the low side to the high side, it could not cross from the high side to the low side with the same driving voltage, because when a droplet was moving from the low side to the high side, the leading edge of the droplet could touch the electrode on the high side with the action of the electrowetting force. On the contrary, when it was moving from the high side to the low side, the electrowetting force was not enough to make the leading edge of the droplet touch the electrode of the lower side, and surface tension of the droplet acted as a key factor. Therefore, a greater voltage was needed to drive the droplet from the height side to the low side.

#### 4.2.3. Effect of Electrode Misalignment of Splicing Chips

As shown in Figure 1b, the electrode misalignment theoretically has a significant influence on the minimum driving voltage of a droplet. In order to investigate this relationship, DMF chips with electrode misalignments of 0, 500, 1000, 1500, and 2000 μm were fabricated, respectively. As shown in Figure 6c, this relationship was verified by experiments. The results indicated that when the electrode misalignment was 500 μm, the minimum driving voltage for crossing the splicing gap hardly changed. When the misalignment was more than 500 μm, the minimum driving voltage was increased with an increase in electrode misalignment. For example, a 10 μL drop could cross a 300 μm splicing gap when the voltage was 119 V, and there was no electrode misalignment. However, when the electrode misalignment was half, the voltage was increased by 32 V, and the theoretical standard error was 7.64%. This error may be caused by the idealization of a droplet in the theoretical calculation, but the actual situation is not so perfect. According to the calculation, a droplet cannot be driven when the electrode misalignment difference is 1440 μm, because there is no electrowetting force when the effect contact line is 0. In an actual driving process, the electrode misalignment was 2500 μm, and a 10 μL droplet could also be driven when the driving voltage was greater than 200 V. Although the electrode misalignment was already large, the droplet area could still cover the misalignment electrode when the droplet was wetted. Therefore, the droplet could be pulled from the previous electrode to the next electrode when the driving voltage was large enough. Then, by repeated tests, a 10 μL droplet could not cross the splicing gap where the electrode misalignment was 3000 μm. As shown in Figure 6d, the video screenshot was displayed when the droplet crossed the splicing gap with a height difference of 150 μm, and as shown in Figure 6e, the video screenshot was displayed when the droplet crossed the splicing gap with an electrode misalignment of 1000 μm, respectively. The electrode on/off time was fixed at 600 ms, and the splicing gap width was fixed at 300 μm.

### 4.3. Effect of Different Aqueous Solutions of the Droplet

**Figure 7:**
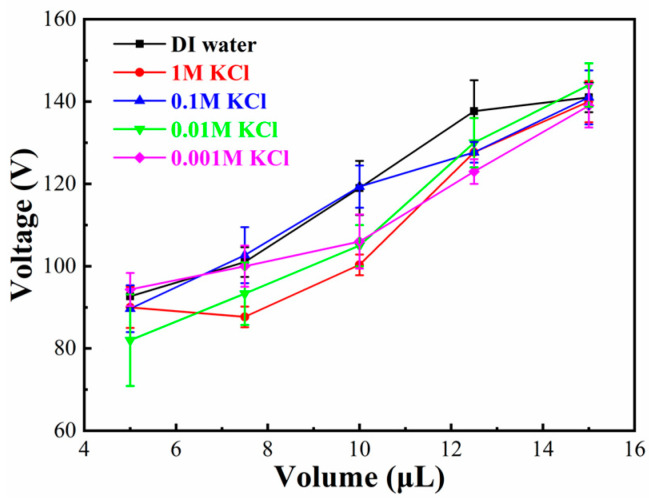
Curves of the relationship between minimum voltage of droplet with different materials and different volumes of droplet.

Due to a wide application of DMF technology, different aqueous solutions of droplets are driven in practical applications. Therefore, deionized (DI) water, 1.0, 0.1, 0.01, and 0.001 M KCl aqueous solutions were driven to cross a splicing gap, respectively. The splicing gap was fixed at 300 μm, the electrode on/off was fixed at 600 ms, and there was no height difference and misalignment difference. As shown in Figure 7, the minimum driving voltage for a droplet with different volumes, which was used to cross the splicing gap, was tested. The droplet volume was bigger, and the minimum driving voltage was greater. The minimum driving voltage for droplets of different materials with the same volume was no obvious voltage change. For instance, the minimum driving voltage for 5 μL droplets of different materials, which was used to cross the same splicing gap, was between 82 and 93 V. Moreover, when the droplet volume was 15 μL, the minimum driving voltage was 139–144 V.

### 4.4. Across Performance Testing of Different Material Substrates

**Figure 8:**
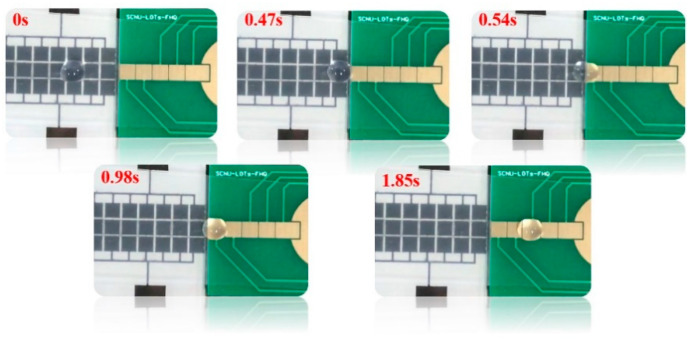
A video screenshot of the droplet which can be driven from the indium tin oxide (ITO) glass DMF chip to the PCB DMF chip.

The substrate material of popular DMF chips includes glass and PCB, etc., and chips of different materials have their own advantages. Therefore, across performance with different substrate materials was tested. The electrode on/off time was fixed at 600 ms, the splicing gap was fixed at 0.1 mm, and the volume of the droplet was 10 μL. The droplet across performance between a chip with a glass substrate and a chip with a PCB substrate was verified. As shown in Figure 8, the result showed that the droplet could cross the splicing gap successfully when the driving voltage was 90 V, and the droplet was successfully driven on the chip with a PCB substrate, what’s more, multiple cycles could be realized (Video S1). Due to the flexibility of the PCB-based DMF chip electrode array design, this schematic demonstrated the powerful potential of DMF chips, which could be applied to complex reactions.

## 5. Conclusions

The splicing of DMF chips was designed using an open modified SLIPS membrane. We investigated the various performance parameters of the droplet for crossing the splicing gap and obtained the relevant change trend. Droplets could be driven between chips with splicing gaps, even if the splicing gap had a height difference and an electrode misalignment, but also could be driven between chips with different substrates. Moreover, the droplets were not affected by the aqueous solutions when they crossed the splicing gap, and the droplet could cross different chip types. Therefore, due to the realization of these solutions, we can carry out multiple tests on human body fluids (sweat, blood, urine, etc.) at the same time to detect human health problems and only one droplet creation module is required.

## Supplementary Materials

The following are available online at https://www.mdpi.com/2072-666X/11/12/1067/s1, Figure S1: Comparison curves of physical representation values with different splicing situations. (**a**) The curve of the relationship between gap width and spacer numbers; (**b**) The curve of the relation between height difference and spacer numbers; (**c**) The curve of the relationship between electrode misalignment and spacer numbers. Video S1: The video of the droplet actuation from ITO-glass chips to PCB chips.
