# Supplementary material for: Designing Splicing Digital Microfluidics Chips Based on Polytetrafluoroethylene Membrane
Source: Micromachines (Basel). 2020 Nov 30;11(12):1067. doi: 10.3390/mi11121067 (PMC7760364; doi:10.3390/mi11121067)
Supplement: Supplementary file 1 [file micromachines-11-01067-s001.zip › micromachines-1015574-supplementary-for final-update/micromachines-1015574-Supplementary Materials-for final.docx]

Supplementary Materials

Designing Splicing Digital Microfluidics Chips Based on Polytetrafluoroethylene Membrane

**Haoqiang Feng, Zichuan Yi, Ruizhi Yang, Xiaofeng Qin, Shitao Shen, Wenjun Zeng, Lingling Shui, Guofu Zhou and Chongfu Zhang**

Photolithography-Etching Process

The electrode array was fabricated on a ITO glass by using a photolithography process. Firstly, the designed electrode array pattern was made into a mask. Secondly, a layer of photoresist (SU-8 2000, Microchem, Round Rock, TX, USA) was applied on the surface of the ITO glass, the spin speed was 2000rpm, the duration was 30s, then, it was baked on hot plate at 100 °C, the duration was 120s. Thirdly, the 365nm UV exposure machine (URE 2000S, China Optoelectronics, China) was used to expose the photoresist-coated ITO glass, the duration was 30s. Fourthly, the pre-configured developer (KOH solution with 5% mass fraction) was used to develop the exposed ITO glass, the duration was 100s, then we took it out and rinsed it with deionized water, the duration was 40s. Next, the film was placed on a hot plate and heated, the hot plate temperature was 120°C, the duration was 30min, and then naturally cooled to room temperature. Fifthly, a solution (H_2_O: concentrated HCl: concentrated HNO_3_ volume ratio was 50:50:3) was used to etch the developed ITO glass. In order to make the etching solution heated evenly, we put the prepared etching solution in warm water with a temperature of 50°C and heated it in a water bath for 3 min. Then, the front of the DMF platform was exposed in the etching solution, and soaked for 90s. The following steps were: rinse the etching solution with deionized water, use alcohol to wash off the photoresist on the chip, use a nitrogen gun to dry.

Characterization of the Splicing Chip

We fabricated the various DMF chips, and the splicing gap width and the electrode misalignment difference were measured by an optical microscope (Axio vert A1, Carl Zesiss AG, Hsu Koehn, Germany), the splicing height was measured by a [step profile](http://dict.youdao.com/w/eng/step%20profile/?spc=step%20profile#keyfrom=dict.typo) (DektaKXT, Bruker, Billica, MA, America). Figure S1 is a comparison chart between theoretical and actual values, we can find that actual values almost completely coincide with theoretical values.

**Figure S1.** Comparison curves of physical representation values with different splicing situations. (**a**): The curve of the relationship between gap width and spacer numbers. (**b**): The curve of the relation between height difference and spacer numbers. (**c**): The curve of the relationship between electrode misalignment and spacer numbers.

**Publisher’s Note:** MDPI stays neutral with regard to jurisdictional claims in published maps and institutional affiliations.

| 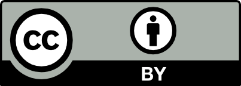 | © 2020 by the authors. Submitted for possible open access publication under the terms and conditions of the Creative Commons Attribution (CC BY) license (http://creativecommons.org/licenses/by/4.0/). |
| --- | --- |
